# Heavy Chains, Heavy Consequences: A Case of Concomitant Heavy Chain Amyloidosis and Heavy Chain Deposition Disease

**DOI:** 10.1016/j.xkme.2025.101102

**Published:** 2025-09-12

**Authors:** Bismark Kojo Amoh, Ghadi Ghanem, Jonathan E. Zuckerman, Zachary Bruss, Kelley Chuang

**Affiliations:** 1 Department of Medicine, David Geffen School of Medicine at the University of California at Los Angeles, Los Angeles, CA; 2 Department of Medicine, Veterans Affairs Greater Los Angeles Healthcare System, Los Angeles, CA

**Keywords:** AH Amyloidosis, heavy chain deposition disease, monoclonal immunoglobulin deposition disease, nephrotic syndrome, monoclonal gammopathy of renal significance

## Abstract

Heavy chain (AH) amyloidosis is a rare form of primary systemic amyloidosis that predominantly affects the kidneys and can lead to nephrotic syndrome. It is marked by the deposition of amyloid fibrils derived from immunoglobulin (Ig)-heavy chains. Monoclonal immunoglobulin deposition disease is a similarly rare disorder involving deposition of nonfibrillar and Congo red-negative monotypic Ig molecules in basement membranes. It can be derived from Ig light chains, Ig heavy chains, or Ig light and heavy chains. Cases of combined amyloidosis and monoclonal immunoglobulin deposition disease are exceedingly rare. Only a handful of concomitant amyloidosis and heavy chain deposition disease have been previously reported, and the spectrum of such diagnoses is poorly described. We describe a case of concomitant IgG4-type AH amyloidosis and heavy chain deposition disease in an 83-year-old man with a history of Agent Orange exposure who developed nephrotic syndrome resulting in diuretic-resistant anasarca. A subsequent bone marrow biopsy demonstrated a λ-restricted plasma cell population. The patient was initiated on hemodialysis and chemotherapy, resulting in clinical stabilization. This case report also contrasts AH amyloidosis with other forms of amyloidosis, emphasizing its unique clinical features, diagnostic challenges, and management considerations.

Systemic amyloidosis is characterized by the extracellular deposition of amyloid fibrils in tissues and organs. Kidney involvement is common and manifests as proteinuria and progressive kidney dysfunction.^1^ Of the types that cause kidney disease, light chain (AL) amyloidosis, involving the deposition of monoclonal immunoglobulin (Ig) light chains, is the most common.^2^ Heavy chain (AH) amyloidosis is a rarer form of primary amyloidosis involving the deposition of heavy chain immunoglobulins that has only been described in case reports.^3^ A similar condition, monoclonal immunoglobulin deposition disease (MIDD), occurs when nonfibrillar, Congo red-negative Ig molecules deposit onto kidney basement membranes. MIDD is classified into 3 subtypes, which include light chain deposition disease, heavy chain deposition disease (HCDD), and light and heavy chain deposition disease. Of these, HCDD and light and heavy chain deposition disease are less common, comprising only 20% of cases.^4^ We report a case of concomitant AH amyloidosis and HCDD presenting as diuretic-resistant nephrotic syndrome.

### Case Report

An 83-year-old man with hyperlipidemia, hypertension, benign prostatic hyperplasia, and Agent Orange exposure presented with 1 week of anasarca, a 20-pound weight gain over 2 months, and progressive dyspnea. Physical examination was significant for pitting edema in his lower extremities up to his sacrum, an S_3_ early diastolic gallop, and decreased breath sounds in his lung bases.

Laboratory studies on admission showed an elevated serum creatinine and findings consistent with nephrotic syndrome, including hypoalbuminemia and nephrotic-range proteinuria (Table 1). The patient had no evidence of cardiac dysfunction or liver failure. His serum and urine protein electrophoresis with immunofixation were positive for an IgG λ monoclonal protein. Free light chain assays demonstrated an elevated λ light chain level. The remaining evaluation for an underlying etiology of nephrotic syndrome was unrevealing (Table 2). His fluid overload was managed with intravenous furosemide. Although his urine output modestly increased, there was no corresponding improvement in his anasarca. A kidney biopsy was performed on hospital day 3. On hospital day 10, the patient developed metabolic acidosis, worsening kidney function, and electrolyte derangements, including hyperkalemia and hyperphosphatemia, which led to hypocalcemia and tetany. He was treated with phosphorus and potassium binders, sodium bicarbonate, and calcium gluconate.

**Table 1:** Laboratory Studies on Admission and Hospital Course

Serum Laboratory Studies	Admission	Hospital Day 10	Hospital Day 18	Reference Range
Creatinine (mg/dL)	1.95 (baseline 1.1)	4.18	7.38	0.6-1.2
Blood urea nitrogen (mg/dL)	18	49	112	6-20
Albumin (g/dL)	2.2	1.8	1.5	3.5-5.0
24-hour urine protein (g/day)	7.4	–	–	<0.15
Bicarbonate (mEq/L)	24.0	21.6	21.2	22-28
Potassium (mEq/L)	4.2	5.5	5.1	3.5-5.0
Phosphate (mg/dL)	3.2	5.5	6.5	2.5-4.5
Calcium (mg/dL)	8.2	7.5	7.0	8.6-10.3
Brain natriuretic peptide (pg/mL)	149	–	–	<100
High-sensitivity troponin (ng/L)	20	–	–	<20
Alanine aminotransferase (U/L)	24	28	26	10-40
Aspartate aminotransferase (U/L)	24	28	13	12-38
Alkaline phosphatase (U/L)	72	72	78	25-100
Serum free light chain concentration (mg/L)	Lambda light chains: 493.7			3.3-19.4
Kappa/lambda ratio	0.07			0.26-1.65

**Table 2:** Laboratory Evaluation for Etiology of Nephrotic Syndrome

Test/Parameter	Result	Reference Range
ANA	Negative	Negative
C3, C4, CH50	Normal	Normal
ANCA, MPO, PR3	Negative	Negative
Anti-PLA2R	Negative	Negative
Hepatitis panel	Negative	Negative
QuantiFERON gold	Negative	Negative

His kidney biopsy was significant for diffuse and global glomerular amyloid deposition confirmed on Congo red stain (Fig 1). Significant interstitial or vascular amyloid deposition was not observed. In addition, there was patchy acute tubular injury, mild tubulointerstitial inflammation, and mild thickening of tubular basement membranes. There was mild interstitial fibrosis, tubular atrophy, and mild arteriosclerosis. Atypical tubular casts were absent. Immunofluorescence stains demonstrated strong smudgy IgG heavy chain staining within the glomerular amyloid deposits and linear staining along glomerular and tubular basement membranes (Fig 1). IgG subclass staining demonstrated IgG4 restriction. There was no significant glomerular or tubular basement membrane staining with either light chain. No light chain restricted casts were present. Significant staining with other reagents (IgA, IgM, C3, C1q, albumin, and fibrinogen) was not present. Electron microscopy revealed the accumulation of nonbranching randomly oriented fibrils within glomeruli and subendothelial and intramembranous powdery electron-dense deposits within glomeruli and tubular basement membranes (Fig 2). No granular, microtubular, or crystalline deposits were present in any compartment. He was diagnosed with concomitant IgG4-type AH amyloidosis and HCDD. Mass spectrometry confirmed amyloidosis of AH (IgG4 heavy chain)-type.

**Figure 1:**
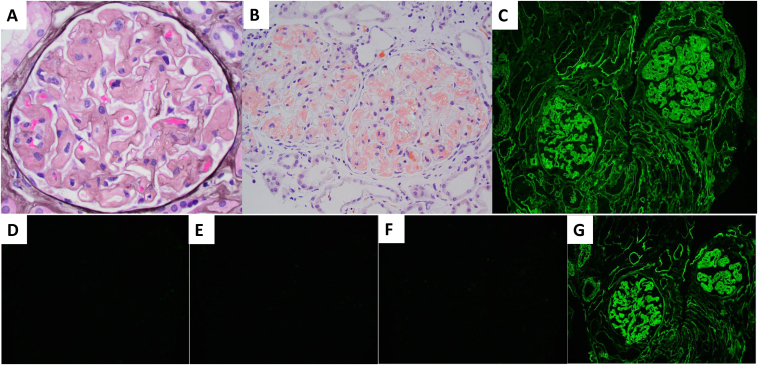
Representative light and immunofluorescence microscopy pathologic findings from the kidney biopsy. (A) Glomerulus globally infiltrated by amorphous acellular non-argyrophilicmaterial (Jones silver stain; Original magnification, ×400) (B) Glomeruli from Congo red stain section demonstrating glomerular congophilia with aberrant colors under polarized light (Original magnification, ×200). Immunofluorescence stains for (C) IgG and IgG heavy chain subclasses (D) IgG1, (E) IgG2, (F) IgG3, and (G) IgG4 demonstrate strong smudgy mesangial, capillary wall, and arteriolar staining as well as linear tubular basement membrane and glomerular capillary wall staining for IgG and IgG4 only.

**Figure 2:**
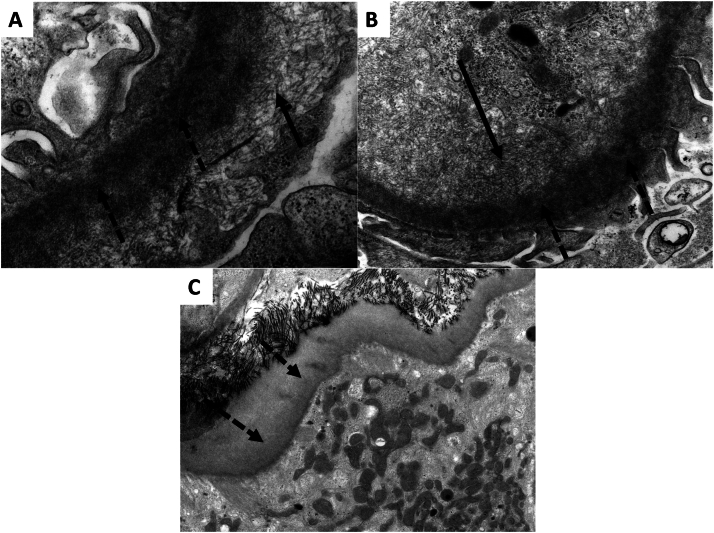
(A-C) Representative electron microscopy findings. High magnification electron micrographs demonstrate accumulation of both nonbranching randomly oriented fibrils (∼11 nm in diameter) (solid arrows) and well as subendothelial and intramembranous powdery electron-dense deposits (dashed arrows). Original magnification (A) ×30,000, (B) ×18,500, and (C) ×6,800.

Bone marrow biopsy to evaluate for other plasma cell dyscrasias revealed a CD56 and CD138 monoclonal plasma cell population that was λ positive and monotypic on flow cytometry. The patient did not meet clinical criteria for multiple myeloma or smoldering myeloma, so was diagnosed with monoclonal gammopathy of renal significance.

Because of the rarity of AH amyloidosis, a treatment strategy based on the established regimen for AL amyloidosis was employed, comprising weekly infusions of daratumumab with cyclophosphamide, bortezomib, and dexamethasone. Medical management of his electrolyte derangements proved challenging, prompting initiation of kidney replacement therapy by hospital day 18. His dyspnea improved with hemodialysis. The patient was discharged with a plan to continue outpatient hemodialysis and chemotherapy infusions.

Two months after hospital discharge, the patient was readmitted with orthostatic hypotension and acute respiratory failure. His symptoms were attributed to dysautonomia secondary to amyloidosis, and residual volume overload. A repeat transthoracic echocardiogram showed a normal ejection fraction of 60%-65%, indicating the absence of cardiac involvement from his amyloidosis. His volume overload improved with hemodialysis, and he was discharged to continue chemotherapy. Approximately 8 months after his index hospitalization, the patient continues to require hemodialysis. He remains on a modified chemotherapy regimen of daratumumab, lenalidomide, bortezomib, and dexamethasone (dara-RVd), with a goal of improving the severe dysautonomia that has significantly limited his daily activities and ability to tolerate hemodialysis.

### Discussion

The rarity of AH amyloidosis is attributed to the lower propensity of immunoglobulin heavy chains to misfold and aggregate into amyloid fibrils.^5^ Systemic amyloidosis is most often caused by AL amyloidosis (up to 85% of cases). Other subtypes of amyloidosis include systemic amyloid A (7% of cases), and leukocyte chemotactic factor amyloidosis (up to 2.5%).^2^ Other rare subtypes of amyloidosis make up the remaining 10% of cases.^4^^,^^5^

The accumulation of amyloid fibrils leads to organ dysfunction due to their insolubility and characteristic β-pleated sheet structure. These fibrils are effectively resistant to proteolytic degradation and cannot be broken down by leukocytes.^6^^,^^7^ In AH amyloidosis, the fibrils are derived from monoclonal Ig heavy chains produced by monoclonal plasma cells. Heavy chains often have structural abnormalities such as truncations or deletions contributing to their amyloidogenic properties. These abnormalities sometimes cause missing epitopes on defective Ig usually targeted by immunohistochemical stains, which can lead to misdiagnosis.^1^^,^^8^ The potential for misdiagnosis may contribute to the low prevalence of AH amyloidosis.

The clinical features of AH amyloidosis stem from the effect of directly cytotoxic amyloid deposits on the charge barrier of glomeruli. This disruption results in the kidney loss of albumin and other proteins, causing fluid buildup in tissues, often manifesting as fatigue and dyspnea. Amyloid deposition on nerve tissue can lead to autonomic dysfunction and orthostatic hypotension, which complicates diuresis. Diuretics are often employed for significant pulmonary edema but are ineffective in reducing amyloidogenic burden. Moreover, they carry the risk of precipitating cast nephropathy due to the accumulation of Bence Jones proteins in the distal nephron, which may further worsen kidney function.^9^

There is currently no standard treatment for AH amyloidosis. Treatment regimens are usually adapted from AL amyloidosis and multiple myeloma with the primary goal of targeting the underlying plasma cell dyscrasia. This is achieved through a combination of chemotherapy agents and can include daratumumab, cyclophosphamide, and bortezomib.^10^ Some patients with AL amyloidosis also undergo autologous stem cell transplantation after chemotherapy, but the efficacy of this method is not established.^11^ Notably, due to a lower incidence of cardiac involvement, patients with AH amyloidosis generally respond better to therapy and have a higher likelihood of complete recovery compared to those with AL amyloidosis.^1^

MIDD exists within a spectrum of diseases that are associated with monoclonal gammopathy of renal significance. Of the 3 subtypes, HCDD is the rarest. In one case series on MIDD, only 7 of 64 patients were diagnosed with the HCDD subtype.^4^ The cause of HCDD is thought to be due to a deficiency in constant region 1 of the heavy chain. The heavy chain therefore fails to bind to a chaperone protein in the endoplasmic reticulum, resulting in plasma cell secretion of a truncated heavy chain. Heavy chains deposit along kidney basement membranes, causing kidney dysfunction. On kidney biopsy, nodular sclerosing glomerulopathy is seen on light microscopy. Diffuse linear staining of the glomerular basement membrane and tubular basement membrane for a single heavy chain is seen on immunofluorescence. Non-fibrillar, powdery deposits on the same basement membranes are seen on electron microscopy.^12^ These characteristic features were found in our patient’s kidney biopsy, along with findings of AH amyloidosis.

The clinical presentation of MIDD varies with each subtype but predominantly causes progressive kidney dysfunction. As with our patient, HCDD can cause nephrotic syndrome from disruption of the glomerular filtration barrier and mesangial expansion by protein deposits. Hypertension, hematuria, and hypocomplementemia are frequently observed.^13^ Concurrent presentation of HCDD and AH amyloidosis likely leads to more rapidly progressive kidney dysfunction.^14^

As with AH amyloidosis, the rarity of HCDD leads to limited knowledge of standard treatment regimens. Treatment options are often extrapolated from treatment of multiple myeloma. Autologous stem cell transplantation may be used after chemotherapy in younger patients with few comorbid conditions to improve hematologic response. Prognostically, a higher baseline serum creatinine is associated with a higher chance of progression to end-stage kidney disease despite treatment. Thus, early detection and treatment lead to better outcomes.^14^

The AH amyloidosis is a rare and complex condition requiring early recognition, comprehensive diagnostics, and a multidisciplinary approach to manage its manifestations effectively. Concurrent diagnosis of AH amyloidosis and MIDD is exceedingly rare. Both conditions individually cause kidney dysfunction; combined, they cause an even more rapidly progressive kidney failure, stressing the importance of early diagnosis and initiation of treatment. Although treatment regimens adapted from AL amyloidosis and multiple myeloma show promise, further research is needed to establish standardized protocols.
